# Zinc triggers a complex transcriptional and post-transcriptional regulation of the metal homeostasis gene *FRD3* in *Arabidopsis* relatives

**DOI:** 10.1093/jxb/erv188

**Published:** 2015-04-21

**Authors:** Jean-Benoit Charlier, Catherine Polese, Cécile Nouet, Monique Carnol, Bernard Bosman, Ute Krämer, Patrick Motte, Marc Hanikenne

**Affiliations:** ^1^Functional Genomics and Plant Molecular Imaging, Center for Protein Engineering (CIP), Department of Life Sciences, University of Liège, B-4000 Liège, Belgium; ^2^Laboratory of Plant and Microbial Ecology, Department of Biology, Ecology, Evolution, University of Liège, B-4000 Liège, Belgium; ^3^Department of Plant Physiology, Ruhr University Bochum, D-44801 Bochum, Germany; ^4^PhytoSYSTEMS, University of Liège, B-4000 Liège, Belgium

**Keywords:** Alternative promoter, *Arabidopsis halleri*, gene regulation, transcript stability, translation, zinc homeostasis.

## Abstract

In *Arabidopsis thaliana*, *FRD3* (*FERRIC CHELATE REDUCTASE DEFECTIVE 3*) plays a central role in metal homeostasis. *FRD3* is among a set of metal homeostasis genes that are constitutively highly expressed in roots and shoots of *Arabidopsis halleri*, a zinc hyperaccumulating and hypertolerant species. Here, we examined the regulation of *FRD3* by zinc in both species to shed light on the evolutionary processes underlying the evolution of hyperaccumulation in *A. halleri*. We combined gene expression studies with the use of β-glucuronidase and green fluorescent protein reporter constructs to compare the expression profile and transcriptional and post-transcriptional regulation of *FRD3* in both species. The *AtFRD3* and *AhFRD3* genes displayed a conserved expression profile. In *A. thaliana*, alternative transcription initiation sites from two promoters determined transcript variants that were differentially regulated by zinc supply in roots and shoots to favour the most highly translated variant under zinc-excess conditions. In *A. halleri*, a single transcript variant with higher transcript stability and enhanced translation has been maintained. The *FRD3* gene thus undergoes complex transcriptional and post-transcriptional regulation in *Arabidopsis* relatives. Our study reveals that a diverse set of mechanisms underlie increased gene dosage in the *A. halleri* lineage and illustrates how an environmental challenge can alter gene regulation.

## Highlight

High expression of the *FRD3* gene in the metal hyperaccumulator *Arabidopsis halleri* stems from complex zinc-regulated transcriptional and post-transcriptional mechanisms present in the non-hyperaccumulating *Arabidopsis thaliana*.

## Introduction

Zinc is an essential micronutrient with numerous important functions in plants but becomes toxic when accumulated in excess (Broadley *et al.*, 2007; Palmer and Guerinot, 2009; Nouet *et al.*, 2011). Plants possess a complex metal homeostasis network that controls the metal supply to tissues throughout development, enabling them to cope with substantial temporal and spatial fluctuations in metal availability in their environment. So-called metal hyperaccumulation, found in approximately 500 plant species, represents a rare and extreme adaptation of the metal homeostasis network. Metal hyperaccumulator plants are able to maintain populations on soils contaminated with highly toxic levels of metals and accumulate extremely high metal concentrations (e.g. >0.3% zinc in leaf dry biomass) in their above-ground tissues (Verbruggen *et al.*, 2009; Krämer, 2010; Hanikenne and Nouet, 2011).

The pseudometallophyte *Arabidopsis halleri* is a zinc and cadmium hypertolerant and hyperaccumulating species. *A. halleri* diverged from *Arabidopsis thaliana* and *Arabidopsis lyrata*, two non-accumulator and non-tolerant species between 3 and 5.8 million years ago and 0.4 and 2 million years ago, respectively (Yogeeswaran *et al.*, 2005; Clauss and Koch, 2006; Roux *et al.*, 2011). These three species represent an ideal experimental model to examine the mechanisms of evolution of a naturally selected extreme trait.

Metal transport, chelation, and detoxification play important roles in hyperaccumulation and hypertolerance in *A. halleri*. About 30 genes involved in these processes are constitutively highly expressed in *A. halleri* when compared with *A. thaliana* and/or *A. lyrata* (Krämer *et al.*, 2007; Verbruggen *et al.*, 2009; Krämer, 2010; Hanikenne and Nouet, 2011). For instance, *HMA4* (*HEAVY METAL ATPASE 4*) is critical for high rates of root-to-shoot translocation of zinc by mediating xylem loading in roots and possibly the intercellular distribution in leaves. High expression of *HMA4* is required for hyperaccumulation and hypertolerance in *A. halleri* (Talke *et al.*, 2006; Courbot *et al.*, 2007; Hanikenne *et al.*, 2008). Increased gene product dosage of *HMA4* was strongly selected for during the evolutionary history of *A. halleri* and evolved through tandem triplication and *cis*-activation of expression of all three gene copies (Hanikenne *et al.*, 2008, 2013). The zinc transporter MTP1 (METAL TOLERANCE PROTEIN 1) is involved in zinc vacuolar storage (Krämer, 2005). MTP1 probably accommodates the high HMA4-dependent metal flux into *A. halleri* shoots and thus would play an important role in zinc tolerance. The *MTP1* gene is constitutively highly expressed in both roots and shoots of *A. halleri* and is present in four to five copies in the *A. halleri* genome (Dräger *et al.*, 2004; Talke *et al.*, 2006; Willems *et al.*, 2007; Shahzad *et al.*, 2010).

Gene copy number variation compared with *A. thaliana* is very likely to contribute to the high expression of many other candidate genes in *A. halleri*, including several *ZIP* (*ZRT-IRT-LIKE PROTEIN*) genes (Talke *et al.*, 2006). At least partially dependent on the activity of HMA4 (Hanikenne *et al.*, 2008), high transcript levels of several *ZIP* genes presumably result in enhanced rates of root metal uptake or mobilization from root storage sites and contribute to metal partitioning between root and shoot tissues (Talke *et al.*, 2006; Krämer *et al.*, 2007; Lin *et al.*, 2009). High expression of the *NAS2* (*NICOTIANAMINE SYNTHASE 2*) gene provides increased nicotianamine levels for intercellular symplastic mobility of zinc towards the xylem in roots (Deinlein *et al.*, 2012).

The *FRD3* (*FERRIC CHELATE REDUCTASE DEFECTIVE 3*) gene is constitutively highly expressed in roots (15-fold) and shoots (6-fold) of *A. halleri* compared with *A. thaliana* (Talke *et al.*, 2006). In *A. thaliana*, *FRD3* encodes a plasma membrane transporter involved in citrate efflux into the xylem (Rogers and Guerinot, 2002; Green and Rogers, 2004; Durrett *et al.*, 2007). Citrate is required for maintaining the apoplastic mobility of iron as a citrate complex in the xylem vessels and in leaf veins (Roschzttardtz *et al.*, 2011). Iron(III)–citrate complexes are responsible for the translocation of an important fraction of iron to shoots (Rellán-Alvarez *et al.*, 2010; Alvarez-Fernández *et al.*, 2014). In *A. thaliana*, *FRD3* is expressed in the root pericycle and vascular cylinder (Rogers and Guerinot, 2002; Green and Rogers, 2004). Mutants of *frd3* are reduced in size and chlorotic, and have reduced citrate and iron contents in xylem sap. They accumulate iron in the root vascular cylinder and the leaf apoplast, accumulate manganese, copper, and zinc in both roots and shoots, and exhibit a constitutive root iron deficiency response (Delhaize, 1996; Rogers and Guerinot, 2002; Green and Rogers, 2004). *FRD3* is also essential during embryo germination and pollen development (Roschzttardtz *et al.*, 2011).

In an analysis of the natural diversity of zinc tolerance across *A. thaliana* accessions, Bay-0 was identified as a zinc-tolerant accession, whereas Shahdara (Sha) was zinc sensitive (Richard *et al.*, 2011). A quantitative trait locus underlying the differential zinc tolerance between Bay-0 and Sha accessions mapped to *FRD3* (Pineau *et al.*, 2012). Differential zinc tolerance among *A. thaliana* accessions was linked to different regulation of *FRD3* expression in response to excess zinc and altered function of the protein. A excess of zinc had a reduced impact on iron homeostasis in the zinc-tolerant Bay-0 accession, suggesting a function of *FRD3* in the connection between zinc and iron homeostasis in *A. thaliana* (Pineau *et al.*, 2012).

Similar to the role of *FRD3* in *A. thaliana*, highly elevated expression of *FRD3* in *A. halleri* may contribute to zinc tolerance and the ability of *A. halleri* to maintain iron homeostasis in zinc hyperaccumulating tissues (Shanmugam *et al.*, 2011). *FRD3* is a single-copy gene in *A. halleri* (Talke *et al.*, 2006). This is in contrast to several other genes with key functions in hyperaccumulation and hypertolerance, of which enhanced expression is, at least in part, caused by gene copy number expansion (see above, and Talke *et al.*, 2006; Hanikenne *et al.*, 2008; Shahzad *et al.*, 2010). To reveal the type of alteration underlying the high expression of *FRD3* in *A. halleri*, we compared the determinants of gene expression of *A. thaliana* and *A. halleri FRD3*. Here, we have shown that promoter activities of *FRD3* genes localize to identical spatial domains in both species, but the genes undergo differential and complex transcriptional and post-transcriptional regulation. In *A. thaliana*, two *FRD3* transcript variants were transcribed from alternative transcription initiation sites. The abundances of the two transcripts were differentially regulated by the zinc supply, favouring a more efficiently translated variant upon zinc excess. Only this latter transcript variant has been retained in *A. halleri*, allowing enhanced translation. Our data highlight that mechanisms other than copy number expansion and *cis*-regulatory changes have contributed to altered metal homeostasis during the evolution of metal hyperaccumulation in *A. halleri*. Moreover, our results contribute a novel aspect to our understanding of the function of *FRD3* in zinc homeostasis of both *Arabidopsis* species.

## Materials and methods

### Plant material, cultivation, and transformation

The experiments were conducted with *A. halleri* ssp. *halleri* (accession Langelsheim) (Talke *et al.*, 2006; Hanikenne *et al.*, 2008), *A. thaliana* [accession Columbia-0 (Col-0)] or zinc-tolerant (Bay-0, NIL-Bay) and zinc-sensitive (Sha, NIL-Sha) *A. thaliana* genotypes (Pineau *et al.*, 2012). Stable transformation of *A. halleri* was performed using a tissue culture-based procedure (Hanikenne *et al.*, 2008). *Nicotiana tabacum* transient transformations were performed by *Agrobacterium* infiltration (Docquier *et al.*, 2004). All metal treatments were conducted under 11h light (100 µmol m^–2^ s^–1^, 22 °C)/13h dark (20 °C) in a climate-controlled growth chamber (Binder) for *A. thaliana* and under 16h light (100 µmol m^–2^ s^–1^)/8h dark at 19–21 °C in a growth chamber for *A. halleri*.

### Plant experimental treatments

Plants were cultivated hydroponically in modified Hoagland’s medium or on solidified Hoagland’s medium supplemented with 0.8% (w/v) agar (Select Agar; Sigma-Aldrich) in square plastic Petri plates (Greiner Bio-One) (Talke *et al.*, 2006; Hanikenne *et al.*, 2008). Control medium included 1 µM Zn for *A. thaliana* and 5 µM Zn for *A. halleri*, and 10 µM Fe^III^-HBED [*N*,*N*’-di(2-hydroxybenzyl)ethylenediamine-*N*,*N*’-diacetic acid monohydrochloride] as the source of iron. Excess zinc (ZnSO_4_.7 H_2_0) was added to the medium as indicated. For zinc deficiency (0 µM Zn) experiments, zinc was omitted from the medium.

*A. thaliana* seeds were germinated on 0.5× MS medium (Duchefa Biochemie) supplemented with 1% (w/v) sucrose and 0.8% (w/v) agar (Select Agar; Sigma-Aldrich). Four days after germination, the seedlings were transferred to Hoagland agar medium to start the treatments. Root and shoot tissues were harvested separately after 17 d treatments.

*A. halleri* individuals were cloned vegetatively by direct rooting in hydroponic medium for 4.5 weeks before initiating experimental treatments. Root and shoot tissues were harvested separately after 3 weeks of treatment.

### *FRD3* promoter cloning

The promoter sequence of the *A. halleri FRD3* gene was identified in an *A. halleri* BAC library (Hanikenne *et al.*, 2008) screened with an *AhFRD3* probe (Talke *et al.*, 2006) as described previously (Benderoth *et al.*, 2006). A total of two positive BAC clones was obtained. BAC DNAs were digested with *Xba*I (a restriction site for *Xba*I is present 317bp downstream of the *AhFRD3* translation initiation codon) and then ligated using T4 DNA ligase. An inverse PCR using primers 5′-CGGCGGTTGGAGTCTCCATTGCCA-3′ and 5′-CGCCGCTAGCTGAGCCGCTCCTAAAC-3′ on the ligation products allowed the amplification of a 2050bp fragment, including the 5′ extremity of the *AhFRD3* coding sequence and 1733bp upstream of the ATG, which was cloned and sequenced. The *AhFRD3* promoter sequences from the two BACs represented two alleles and were 97% similar, with the exception of a 155bp insertion in one allele.

### Cloning

5′ Rapid amplification of cDNA ends (RACE) was conducted using a SMART RACE cDNA amplification kit (Clontech). All *FRD3* sequences were PCR amplified (see Supplementary Table S1 available at *JXB* online for primer sequences) using a proofreading polymerase (*Pfu* polymerase, Promega; and Bio-X-ACT Long, Bioline) and verified by sequencing.

The *AhFRD3* promoter sequence was amplified from genomic DNA of the LAN3.1 genotype of *A. halleri* ssp. *halleri* (Hanikenne *et al.*, 2008). The AtFRD3_Full_ (4506bp) and AtFRD3_Trunc_ (2220bp) promoter fragments were amplified from genomic DNA of *A. thaliana* (Col-0). All promoter fragments, which included the promoter, 5′-untranslated region (5′UTR) and the first 27bp of the respective *FRD3* coding sequence were directionally cloned into pENTR/D TOPO (Invitrogen) and then transferred by Gateway recombination into the pMDC163 binary vector in translational fusion with the β-glucuronidase (*GUS*) gene (Curtis and Grossniklaus, 2003).

**Fig. 1.**
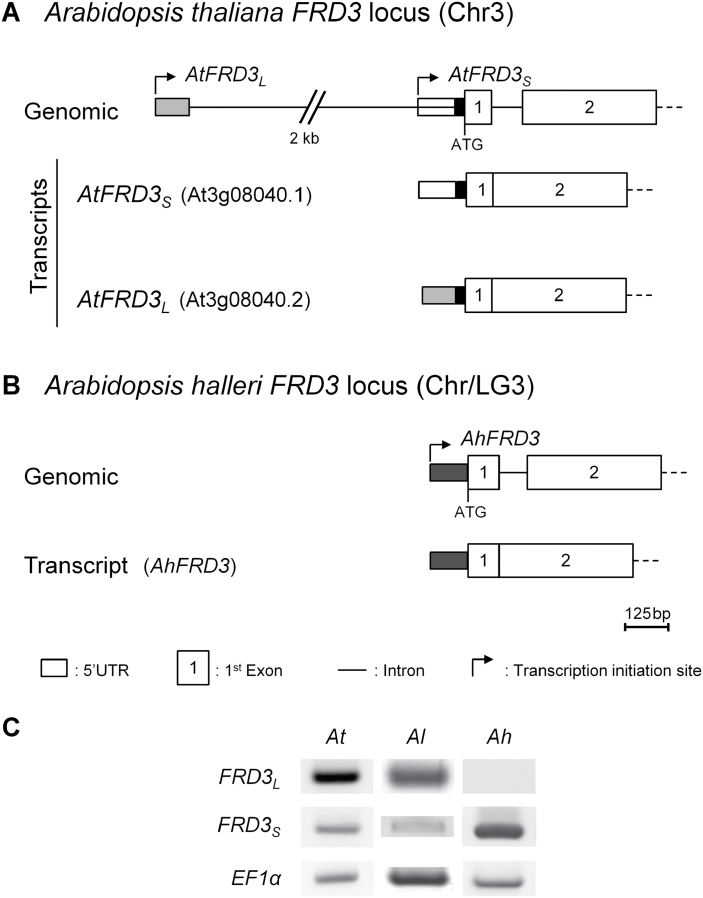
Transcription initiation sites of *FRD3* genes. (A, B) Organization of the *FRD3* genomic loci and transcripts in *A. thaliana* (A) and *A. halleri* (B). For narrow rectangles denoting the 5′UTR, distinct white, grey shading, or black represent alternative segments of the *FRD3* sequence. (C) *FRD3* transcript variants (*FRD3*_*L*_ and *FRD3*_*S*_) were detected with specific primers by qualitative RT-PCR in shoots of *A. thaliana* (*At*), *A. lyrata* (*Al*) and *A. halleri* (*Ah*). *EF1α* was used as a control.

The 5′UTR_L_ (89bp) and 5′UTR_S_ (102bp) of *AtFRD3*, and the 5′UTR (103bp) of *AhFRD3* (see Fig. 1) were amplified from *A. thaliana* and *A. halleri* cDNA libraries, respectively. 5′UTR fragments were fused to the green fluorescent protein (GFP) coding sequence using a *Kpn*I restriction site included in the primer sequences (Supplementary Table S1), and the 5′UTR–GFP cassettes were subcloned at the *Asc*I and *Pac*I sites of the pMDC32 binary vector downstream from the 35S promoter (Curtis and Grossniklaus, 2003). *GFP* alone and the luciferase gene (*LUC*) alone were inserted at the *Asc*I and *Pac*I sites of pMDC32 to generate controls.

### Analysis of GUS reporter lines

Histochemical GUS staining (Jefferson *et al.*, 1987) was carried out on T3 homozygous *A. thaliana* seedlings grown on 0.5× MS medium, and on *A. halleri* transformants regenerated in tissue culture under selection. Transverse sections of tissues were prepared and imaged as described previously (Hanikenne *et al.*, 2008). Ten independent lines were analysed for each construct for whole mounts, and at least three representative lines for cross-sections. Fluorimetric quantitative GUS activity assays were performed as described elsewhere (Jefferson *et al.*, 1987; Hanikenne *et al.*, 2008).

### Gene expression analyses

Total RNAs were prepared using an RNeasy Plant Mini kit with on-column DNase treatment (Qiagen), and cDNAs were synthesized using a RevertAid H Minus First Strand cDNA Synthesis kit with Oligo dT (Thermo Scientific). Steady-state transcript levels were determined following a robust reverse transcription (RT)-PCR procedure (see Supplementary Table S2 available at *JXB* online for primer sequences). Briefly, after PCR amplification with GoTaq polymerase (Promega), PCR products were separated in a 1.5 % LE agarose gel (SeaKem; Lonza) in sodium borate buffer (Brody *et al.*, 2004). As an inter-run calibrator, a common DNA fragment was loaded on each gel, enabling gel-to-gel comparisons. After gel staining with GelRed (Biotium), the gels were imaged with identical settings using a G:BOX imaging system (Syngene), and absolute band intensities were determined in Photoshop (Adobe Systems Software). Expression values were normalized to *EF1α* and the inter-run calibrator. Relative *AtFRD3*_*S*_ steady-levels were calculated as a percentage of total *AtFRD3* transcripts using the following formula: *AtFRD3*_*S*_/(*AtFRD3*_*S*_+*AtFRD3*_*L*_)×100.

### Analysis of metal contents

Root and leaf tissues were harvested separately. Shoot tissues were rinsed in Milli-Q water, whereas root tissues were desorbed and washed as described previously (Talke *et al.*, 2006). Dried tissue samples were acid- digested in DigiPrep tubes with 3ml of ≥65% (w/w) HNO_3_ (Sigma-Aldrich) on a DigiPrep Graphite Block Digestion System (SCP Science) as follows: 15min at 45 °C, 15min at 65 °C, and 90min at 105 °C. After cooling, sample volumes were adjusted to 10ml with Milli-Q water and 200 µl of ≥65% HNO_3_. Element concentrations were determined by inductively coupled plasma-atomic emission spectroscopy (Vista AX, Varian).

### Cordycepin assay

Cordycepin assays (Gutiérrez *et al.*, 2002) were conducted with 12-d-old *A. thaliana* seedlings, *A. halleri* root fragments of 3- to 4-week-old plants, and fragments of tobacco leaf transiently transformed with the genes of interest. Samples were first incubated in Hoagland control medium for 30min at room temperature under slight shaking in the light. At time 0 (T0), samples were vacuum infiltrated with Hoagland control medium containing 100 µM cordycepin (Sigma-Aldrich) for 30 s and then incubated at room temperature in the light. Subsamples (10 *A. thaliana* seedlings or *A. halleri* root fragments or five tobacco leaf explants) were harvested at regular time intervals.

Transcript levels were determined by real-time RT-PCR in 384-well plates with an ABI Prism 7900HT system (Applied Biosystems) using MESA GREEN qPCR MasterMix (Eurogentec) as described previously (Talke *et al.*, 2006), including three technical replicates for each sample/primer pair (see Supplementary Table S3 available at *JXB* online for primer sequences). Transcript levels were normalized to T0 and fitted by non-linear regression according to the following exponential decay formula: *Y*=(*Y*_0_–plateau)×exp(–K×*X*)+plateau with fixed parameters (plateau >0, *Y*_0_=1, *K*>0). Plateau is the *Y* value at infinite times, K is the rate constant expressed in inverse minutes, and *Y*_0_ is the *Y* value when *X* (time) is 0. Half-life was then computed as ln_2_/K. To test statistical differences between estimated half-life, we followed this non-linear regression by an extra-sum-of-squares F comparison test.

### GFP quantification and imaging

At 3–4 d after transient co-transformation with GFP and LUC constructs, tobacco leaf fragments (1cm^2^) were cut off either for protein extraction or for confocal imaging. Total proteins were extracted from leaf fragments using the CCLR buffer of a Luciferase Assay System kit (Promega). GFP was quantified in protein extracts by fluorimetry (excitation at 485nm and emission at 535nm) using a Victor 3 plate spectrophotometer (Perkin Elmer). GFP fluorescence levels were normalized to LUC activity (Petit *et al.*, 2001) determined using the Luciferase Assay System kit and a tube luminometer (Lumat LB 9501, Berthold).

GFP was imaged by confocal microscopy ensuring that the maximal fluorescence signal was not saturating the photomultiplier tubes, as described previously (Rausin *et al.*, 2010). For GFP quantification, *Z*-optical sections of 45 and 65 µm were taken in randomly selected zones of leaf fragments. The resulting images were then stacked and three regions of interest were selected in each visible nucleus. GFP signal intensity was measured as the mean grey intensity in each region of interest, and the mean grey intensity for each construction was then computed.

### Secondary structure prediction

The structure and minimum free energies of the mature *AtFRD3*_*L*_ and *AtFRD3*_*S*_ full-length transcripts were calculated using Centroidfold with default settings (Sato *et al.*, 2009).

### Statistical analysis

All data evaluation and statistics were done using GraphPad Prism 5 (GraphPad Software).

### Accession numbers

The sequence of the *AhFRD3* promoter is available through EBI (http://www.ebi.ac.uk), accession no. Hx2000040684. The *AtFRD3* TAIR accession number is At3g08040 (http://www.arabidopsis.org).

## Results

### Alternative transcription initiation sites

In *A. thaliana*, two *FRD3* transcript variants, At3g08040.1 and At3g08040.2 (The *Arabidopsis* Information Resource, http://www.arabidopsis.org), possess distinct transcription initiation sites (Fig. 1A). We verified the transcription initiation sites and expression of the two transcript variants in *A. thaliana* by 5′RACE. A short transcript (*AtFRD3*_*S*_ or At3g08040.1) was initiated 102bp upstream of the AUG translation initiation codon (Fig. 1A). The long transcript (*AtFRD3*_*L*_ or At3g08040.2) was initiated 2726bp upstream of the AUG. The corresponding pre-mRNA contained a large intron (2637 nt) in the 5′UTR. The mature transcript possessed a 5′UTR of 89 nt (Fig. 1A). The *AtFRD3*_*L*_ and *AtFRD3*_*S*_ transcripts shared 27 nt upstream of the AUG. Out of 17 independently sequenced 5′RACE clones, 13 corresponded to *AtFRD3*_*L*_ and four to *AtFRD3*_*S*_.

In *A. halleri*, 5′RACE detected a single transcript (*AhFRD3*) corresponding to the short variant of *A. thaliana*, out of 12 independent clones sequenced, with a 103bp 5′UTR not spanning an intron (Fig. 1B). *AtFRD3*_*S*_ and *AhFRD3* shared an evolutionary conserved transcription initiation site and their 5′UTRs were 95% identical.

Variant-specific primers were designed based on *A. thaliana* sequences, which allowed the detection of two *FRD3* transcript variants in both *A. thaliana* and *A. lyrata* (Fig. 1C). Again, a single transcript variant was detected in *A. halleri* (Fig. 1C). This suggests that the presence of two *FRD3* transcript variants is the ancestral state in the genus *Arabidopsis*, whereas a single transcript in *A. halleri* corresponds to the derived state (Supplementary Fig. S1, available at *JXB* online). *In silico* analyses of available genomic sequences for *FRD3* orthologues suggested the presence of two *FRD3* transcript variants in other Brassicaceae, with either the annotation of two variants (*Boechera stricta*) or sequence conservation at splicing and transcript initiation sites (*Capsella rubella*, *Brassica rapa*, and *Eutrema halophilum*) (http://www.phytozome.net).

### Conserved expression profiles

The most detailed analysis of the *AtFRD3* expression profile was reported by Roschzttardtz *et al.* (2011) using a *GUS* reporter construct that included 1751bp upstream of the ATG as promoter. This reporter construct included the proximal site of transcription initiation only (Fig. 1A, Supplementary Fig. S2 available at *JXB* online). To determine the expression profile of an *AtFRD3* promoter sequence that included both transcription initiation sites, and to examine whether the expression profiles of *FRD3* are conserved in *A. thaliana* and *A. halleri*, we generated fusions to the *GUS* reporter. Three constructs were made using full-length (4506bp, pAtFRD3_Full_) and truncated (2220bp, pAtFRD3_Trunc_) *AtFRD3* promoters, including both transcript initiation sites and the proximal site alone, respectively, and the *AhFRD3* promoter (1885bp) (Supplementary Fig. S2). The pAtFRD3 promoter fragments shared 88% sequence identity with pAhFRD3 in the first 269bp upstream from the ATG. Upstream from this proximal fragment, sequence divergence precluded an alignment.

**Fig. 2.**
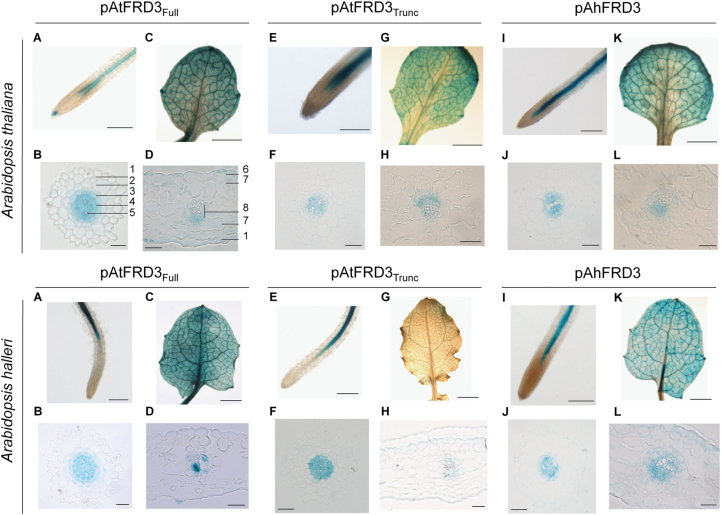
Localization of *FRD3* promoter activity in *A. thaliana* and *A. halleri*. Histochemical detection of GUS activity (blue) directed by a full (pAtFRD3_Full_) (A–D) and a truncated (pAtFRD3_Trunc_) (E–H) *A. thaliana FRD3* promoter, or the *A. halleri FRD3* (pAhFRD3) promoter (I–L) in whole mounts (A, C, E, G, I, K) and transverse sections (B, D, F, H, J, L) of roots (A, B, E, F, I, J) and leaves (C, D, G, H, K, L) of 3-week-old *A. thaliana* (top) and *A. halleri* (bottom) plants. Note that only weak GUS staining was observed for pAtFRD3_Trunc_ in *A. halleri* shoots. Bars, 1.5mm (A, E, I), 5mm (C, G, K) and 25 µm (B, D, F, H, J, L). 1, epidermis; 2, cortex; 3, endodermis; 4, pericycle; 5, xylem; 6, stomata; 7, mesophyll, 8, vascular bundle.

Spatial patterns of reporter activity for all three constructs were highly similar in both *A. thaliana* and *A. halleri* (Fig. 2). The *FRD3* promoters were active in the root pericycle and vascular cylinder in both species, as described for the *A. thaliana* gene (Green and Rogers, 2004; Roschzttardtz *et al.*, 2011), and in leaves, mostly in vascular tissues, in hydathodes and in mesophyll cells (Fig. 2). Only a slight GUS coloration could be detected for pAtFRD3_Trunc_ in *A. halleri* leaves (Fig. 2). Moreover, two *GUS* transcripts were initiated from the full *AtFRD3* promoter in reporter lines of both species (Supplementary Fig. S3 available at *JXB* online).

**Fig. 3.**
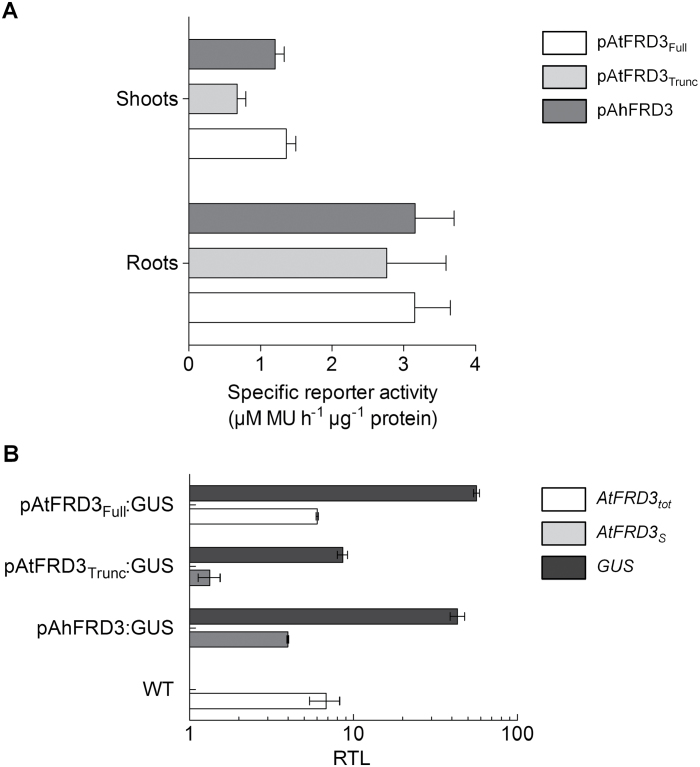
*FRD3* promoter activity in *A. thaliana*. (A) Specific GUS activity was quantified in total protein extracts from roots and shoots of *A. thaliana* seedlings expressing the *GUS* reporter gene under the control of a full-length (pAtFRD3_Full_) and a truncated (pAtFRD3_Trunc_) *A. thaliana FRD3* promoter, or the *A. halleri FRD3* (pAhFRD3) promoter. Homozygous seedlings from four independent lines for each construct (T3 generation) were grown on solidified control Hoagland medium. Roots and shoots were harvested separately and pooled per plate (15 seedlings), with two replicate Petri plates per line. Values are mean± SEM with *n*=4 independent lines from one experiment representative of two independent experiments. MU, 4-methylumbelliferone. (B) Expression analysis of the endogenous *AtFRD3* and *GUS* genes in *A. thaliana* GUS reporter lines. Steady-state levels of *GUS*, total *AtFRD3* (*AtFRD3*_*tot*_), and short *AtFRD3* (*AtFRD3*_*S*_) transcripts were determined by real-time RT-PCR in 12-d-old *A. thaliana* seedlings expressing the *GUS* reporter gene under the control of a full-length (pAtFRD3_Full_) and a truncated (pAtFRD3_Trunc_) *A. thaliana FRD3* promoter, or the *A. halleri FRD3* (pAhFRD3) promoter. Values are means of three technical replicates of one representative line for each construct grown on solid Hoagland control medium. RTL, relative transcript level.

Quantitative analysis of GUS activity in protein extracts from *A. thaliana* seedlings suggested that the three promoters were more active in roots than in shoots. The pAhFRD3 promoter mediated high *GUS* transcript expression and high GUS activity in *A. thaliana* (Fig. 3), which reflected the high expression of *FRD3* in *A. halleri* (Talke *et al.*, 2006). This suggested a conserved regulation *in cis* for high expression of *AhFRD3* in *A. thaliana* and *A. halleri*. Surprisingly, the two pAtFRD3 promoter fragments mediated equally high activity of the reporter protein (Fig. 3A). We quantified both *GUS* and endogenous *AtFRD3* transcript levels in reporter lines and observed that GUS activity did not reflect the expression of the endogenous *AtFRD3* gene in these plants (Fig. 3B). Indeed, *GUS* transcript levels were significantly higher than: (i) the total *AtFRD3* transcript levels (*AtFRD3*_*tot*_) in pAtFRD3_Full_ lines expressing both long and short *GUS* transcript variants (Supplementary Fig. S3), and (ii) *AtFRD3*_*S*_ transcript levels in pAtFRD3_Trunc_ lines, expressing only the short *GUS* transcript variant (Supplementary Fig. S3) (Fig. 3B).

### Differential gene regulation by zinc

So far, little evidence is available supporting a metal-dependent regulation of the *FRD3* gene expression in *A. thaliana* or *A. halleri* (Rogers and Guerinot, 2002; Talke *et al.*, 2006). We further examined the transcriptional regulation of *FRD3* by zinc in root and shoot tissues of *A. thaliana*. Seedlings were exposed to zinc deficiency or to moderate zinc excess. Under these growth conditions, zinc concentrations in plant tissues reflected metal supply in the medium (Supplementary Fig. S4 available at *JXB* online) and the zinc-responsive gene *AtZIP4* was strongly upregulated in both roots and shoots upon zinc deficiency (Supplementary Fig. S5 available at *JXB* online), as expected (Talke *et al.*, 2006). The zinc treatments did not alter iron concentrations in the seedlings (Supplementary Fig. S4) or *AtIRT1* expression (data not shown).

**Fig. 4.**
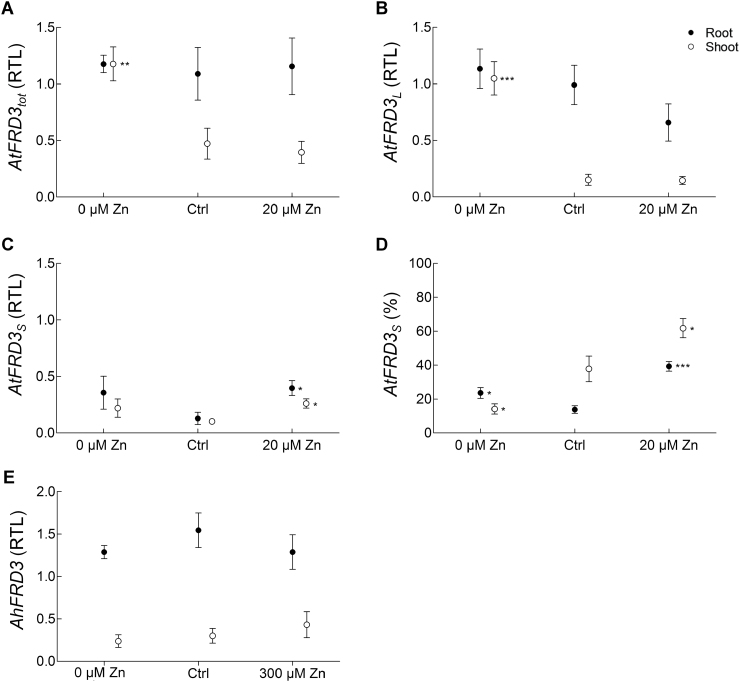
Dependence of transcript abundance of *FRD3* variants on zinc supply in *A. thaliana* and *A. halleri*. Steady-state transcript levels for total *AtFRD3* (*AtFRD3*_*tot*_) (A), *AtFRD3*_*L*_ (B), *AtFRD3*_*S*_ (C), and *AtFRD3*_*S*_ (D) expressed as a percentage of total *AtFRD3* transcript levels and for *AhFRD3* (E). Steady-state transcript levels were determined in the roots and shoots of *A. thaliana* and *A. halleri* cultivated under control conditions (Ctrl), upon zinc deficiency (0 µM Zn) and zinc excess (20 µM Zn for *A. thaliana* and 300 µM Zn for *A. halleri*). Values were normalized to *EF1α* and an inter-run calibrator. The inter-run calibrator differed for each species, and thus transcript levels could only be compared within species. Values are means±SEM of four (A–D) or two (E) independent experiments. Independent experiments included pools of at least 25 *A. thaliana* seedlings grown on Hoagland agar medium plates (A–D) or six *A. halleri* plants grown hydroponically in Hoagland medium (E) for each condition. *P<0.05, **P<0.01, and ***P<0.001 according to one-way analysis of variance, followed by Dunnett’s test for multiple comparisons of means. RTL, relative transcript level.

Upon growth under control conditions, *AtFRD3*_*tot*_ transcript levels were lower in shoots than in roots (Fig. 4A). Transcript levels of *AtFRD3*_*tot*_ in roots did not respond to the zinc supply in the medium, whereas they were significantly increased in shoots upon zinc deficiency (Fig. 4A).

We next examined changes in the levels of the two *AtFRD3* transcript variants in response to zinc status. Under control conditions, *AtFRD3*_*S*_ was the minor form, representing ~14% of *AtFRD3* transcripts in roots and ~40% in shoots, respectively (Fig. 4D). In both roots and shoots, the levels of *AtFRD3*_*S*_ increased upon zinc excess (Fig. 4C), whereas *AtFRD3*_*L*_ levels were unchanged (Fig. 4B). The differential regulation of the two transcripts by zinc excess significantly modified the *AtFRD3*_*S*_/*AtFRD3*_*L*_ ratio, with *AtFRD3*_*S*_ representing ~40% of the total transcripts in roots and becoming the dominant form in shoots (~62%) (Fig. 4D). In contrast, *AtFRD3*_*L*_ was strongly induced by zinc deficiency in shoots (Fig. 4B), and this induction accounted for the increased levels of *AtFRD3*_*tot*_ transcripts (Fig. 4A).

For comparison, in hydroponically grown vegetative *A. halleri* plants, no significant zinc-dependent changes in *AhFRD3* transcript levels were observed (Fig. 4E), whereas *AhZIP4* transcript levels responded to zinc deficiency or excess (Supplementary Fig. S5), as expected (Talke *et al.*, 2006). Zinc excess resulted in increased root iron contents, but reduced shoot iron levels (Supplementary Fig. S4).

### Transcript stability

**Fig. 5.**
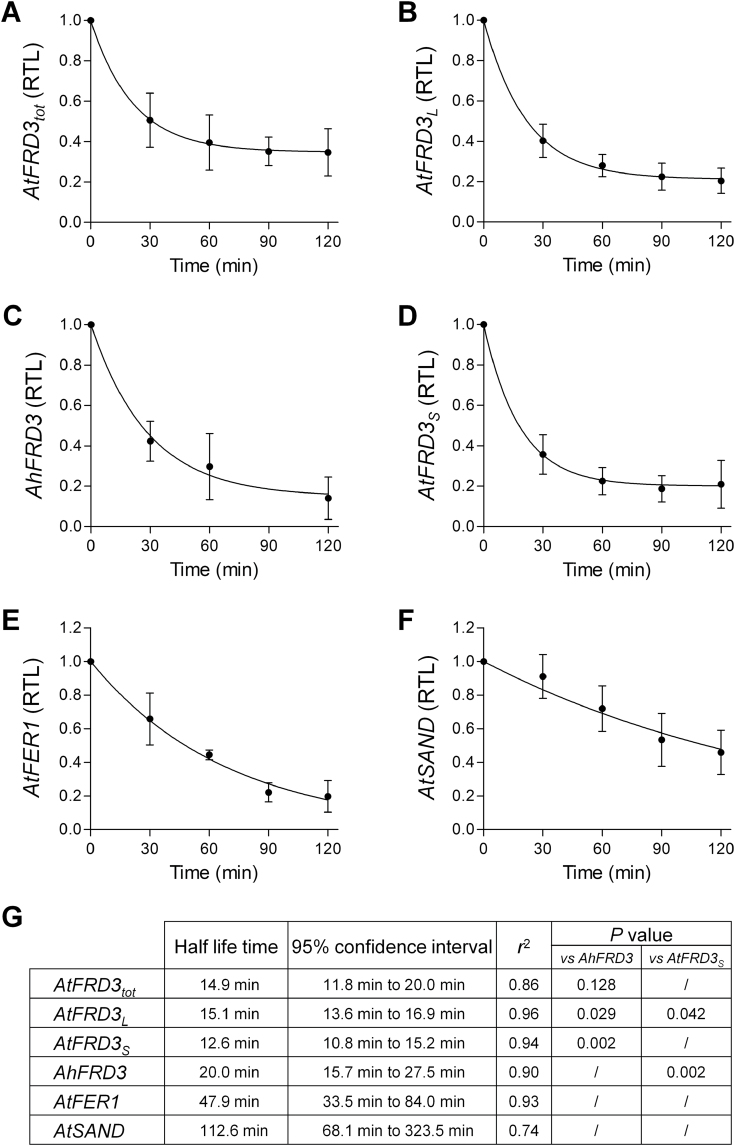
*FRD3* transcript stability. (A–F) The half-life times of total *AtFRD3* (*AtFRD3*_*tot*_) (A), *AhFRD3* (B), *AtFRD3* long (*AtFRD3*_*L*_) (C), and short (*AtFRD3*_*S*_) (D) transcripts were determined in the presence of the transcriptional inhibitor cordycepin, with *AtFER1* (E) and *AtSAND* (F) included as controls (Ravet *et al.*, 2012). Seedlings (*A. thaliana*) and root segments (*A. halleri*) were collected at several time points after the onset of cordycepin treatment. Transcript levels were determined by real-time RT-PCR. Values were normalized to time 0 and fitted by non-linear regression. (G) Transcript half-life (in min), confidence interval at 95%, regression correlation for the fit of the curve (*r* ^2^) and *P* values of an extra-sum-of-squares F comparison test for statistical differences between estimated half-life. Values are means±SEM of nine (A, C, D, E, F) or four (B) independent experiments. Each independent experiment included at least three technical replicates. RTL, relative transcript level.

In *A. thaliana* and *A. halleri*, *AtFRD3*_*tot*_ and *AhFRD3* transcripts accumulated at different steady-state levels, respectively. In *A. thaliana*, steady-state levels of the *AtFRD3*_*L*_ and *AtFRD3*_*S*_ transcript variants were also distinct. These differences could result from differences in either the rates of transcription or RNA stability. To discriminate between these alternative hypotheses, we determined the half-life time of *FRD3* transcripts using cordycepin, an inhibitor of transcription, in *A. thaliana* seedlings and in roots of vegetative *A. halleri* plants. The half-life of *AhFRD3* transcripts (20.0min) was slightly, but not significantly, longer than that of overall *AtFRD3*_*tot*_ transcripts (14.9min; Fig. 5). Moreover, the half-life of both *AtFRD3* transcript variants was similar, with 12.6min for *AtFRD3*_*S*_ and 15.1min for *AtFRD3*_*L*_ (Fig. 5). The *FRD3* transcripts could be considered as relatively unstable, compared with the half-life of control genes (47.9 and 112.6min for *AtFER1* and *AtSAND*, respectively; Fig. 5) (Ravet *et al.*, 2012). These data suggested that differences in steady-state transcript levels of *AtFRD3*_*tot*_ and *AhFRD3* on the one hand, and of *AtFRD3*_*L*_ and *AtFRD3*_*S*_ on the other hand, were mostly stemming from differential transcription initiation rates. In contrast, *AtFRD3*_*S*_ and *AhFRD3* displayed more substantial differences in transcript stabilities (~1.6-fold), which may contribute to overall differences in *FRD3* transcript levels in the two species (Fig. 5G).

### Differential translation efficiency

The two *AtFRD3* transcript variants differed in their 5′UTR but were strictly identical in their coding sequences. This raised the question of the functional significance of the complex regulation of *AtFRD3*. We next tested the hypothesis that the alternative 5′UTRs alone had an impact on post-transcriptional processes using a reporter system (Dvir *et al.*, 2013; Kim *et al.*, 2014). To this end, the *AtFRD3*_*L*_ and *AtFRD3*_*S*_ 5′UTRs were cloned upstream of the *GFP* coding sequence, placed under the control of the cauliflower mosaic virus 35S promoter and transiently expressed in tobacco leaves. A similar construct was generated with the *AhFRD3* 5′UTR to allow comparison.

**Fig. 6.**
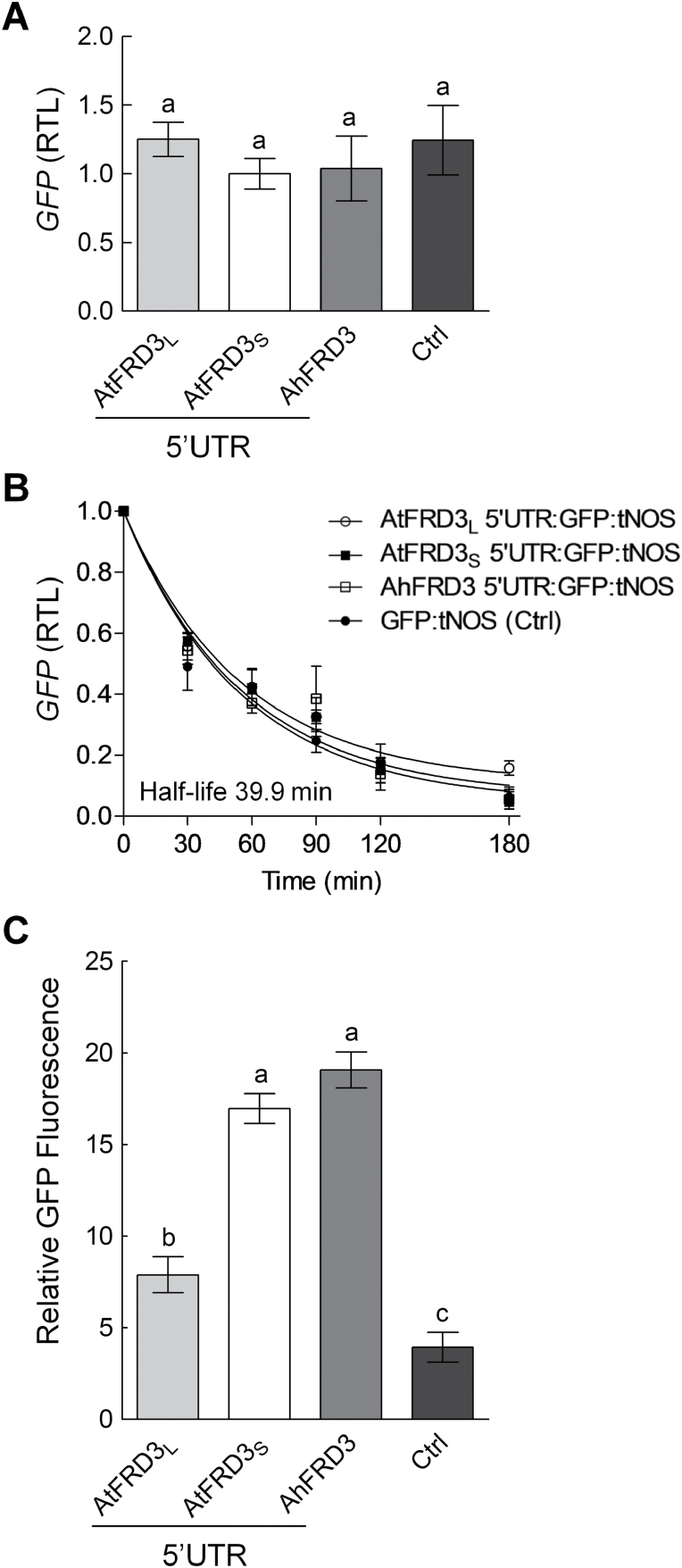
Contributions of the 5′UTR of *FRD3* transcript variants to steady-state transcript levels, transcript stability, and levels of the encoded protein. The *AtFRD3*_*L*_, *AtFRD3*_*S*_, and *AhFRD3* 5′UTR fused to the *GFP* coding sequence were transiently expressed in tobacco leaves by *Agrobacterium* infiltration under the control of a 35S promoter. A 35S:GFP control (Ctrl) lacking a 5′UTR was included in the experimental design. Leaf fragments were harvested 3–4 d post-infiltration. (A) Steady-state *GFP* transcript levels were normalized to *HygB* transcript levels generated *in vivo* from the introduced T-DNA and are given relative to *AtFRD3*_*S*_ samples. (B) *GFP* transcript stability (cordycepin assay). Infiltrated leaf fragments were collected at several time points after the onset of cordycepin treatment. GFP transcript levels were determined by real-time RT-PCR. Values were normalized to time 0 and fitted by non-linear regression. (C) Quantification of GFP protein levels in total protein extracts by fluorimetric assay normalized to LUC activity. A 35S:LUC construct was co-infiltrated with all GFP constructs. Different letters above bars indicate significantly different values (*P*<0.05) according to a *t*-test. Values are means±SEM of two (A, C) or three (B) independent experiments, respectively. Independent experiments included at least three leaf fragments in three technical replicates. RTL, relative transcript level.

Relative abundances of *GFP* transcripts and their stabilities (half-life of 39.9min) were identical for all three constructs and for a 35S:GFP control (Fig. 6A, B), indicating that the 5′UTRs of *FRD3* do not regulate transcriptional processes and transcript stability. In contrast, GFP protein levels were ~2.5-fold higher in tissues transformed with the *AtFRD3*_*S*_ and *AhFRD3* 5′UTRs by comparison with the *AtFRD3*_*L*_ 5′UTR, respectively (Fig. 6C). Qualitative analyses *in vivo* using confocal microscopy yielded comparable results (Supplementary Fig. S6 available at *JXB* online). These data suggested that *FRD3* 5′UTRs specify differential translation efficiencies.

As secondary structures are known to influence translation efficiency (Bugaut and Balasubramanian, 2012; Pichon *et al.*, 2012), we used the Centroidfold algorithm (Sato *et al.*, 2009) to predict the secondary structures of the full-length *AtFRD3* transcript variants (Supplementary Fig. S7 available at *JXB* online). Globally, the two structures were very similar, but a disorganized loop at the 5′ extremity of *AtFRD3*_*L*_ (Supplementary Fig. S7A) was absent in *AtFRD3*_*S*_ (Supplementary Fig. S7B).

### *AtFRD3* regulation in zinc-tolerant and zinc-sensitive accessions

**Fig. 7.**
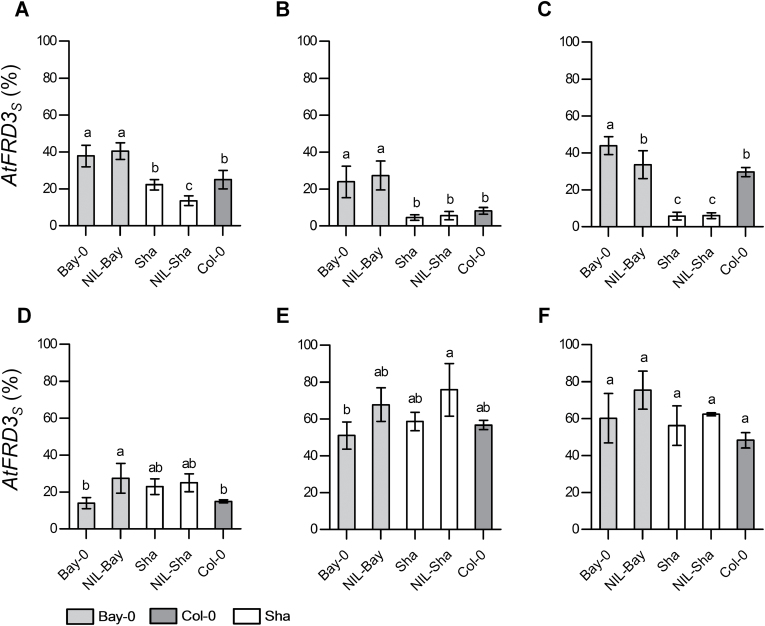
Differential transcriptional regulation of *FRD3* in zinc-tolerant and zinc-sensitive *A. thaliana* genotypes. Steady-state transcript levels were determined for the *AtFRD3* short (*AtFRD3*_*S*_) transcript in roots (A–C) and shoots (D–F) of 21-d-old *A. thaliana* seedlings grown under control conditions (Ctrl, 1 µM Zn) (B, E), under zinc deficiency (0 µM Zn) (A, D), and under zinc excess (20 µM Zn) (C, F) for 17 d. Bay-0 and NIL-Bay are zinc-tolerant genotypes, whereas Sha and NIL-Sha are zinc-sensitive genotypes (Pineau *et al.*, 2012). Col-0 was included as reference genotype. *AtFRD3*_*S*_ levels are expressed as a percentage of total *AtFRD3* transcripts (see Materials and methods). Total steady-state *AtFRD3* transcript levels are given in Supplementary Fig. S8. Values were normalized to *EF1α* and an inter-run calibrator. Values are means±SEM of two independent experiments. Each independent experiment included at least 25 pooled seedlings per treatment and genotype. Different letters above histogram bars indicate significantly different values (*P*<0.05) within treatments according to a one-way analysis of variance followed by Dunnett’s test for multiple comparison of means.

We next examined whether the transcriptional regulation of *AtFRD3*_*tot*_ and the *AtFRD3*_*L*_ and *AtFRD3*_*S*_ transcript variants by zinc differed in zinc-sensitive and zinc-tolerant *A. thaliana* genotypes. To do so, we made use of the Bay-0 and Sha accessions, as well as two near-isogenic lines, NIL-Bay and NIL-Sha, expressing the *FRD3* ^*Bay*^ gene in a Sha genetic background and vice versa (Pineau *et al.*, 2012). In roots, *AtFRD3*_*tot*_ transcript levels were unchanged by zinc in all genotypes including Col-0 (Supplementary Fig. S8 available at *JXB* online). In contrast, steady-state levels of *AtFRD3*_*S*_ were constitutively higher in roots of zinc-tolerant genotypes (Bay-0 and NIL-Bay) than in Col-0 in control conditions and were only increased slightly in response to zinc treatments (Fig. 7). In zinc-sensitive genotypes (Sha and NIL-Sha), steady-state levels of *AtFRD3*_*S*_ were similar to Col-0 in roots under control conditions. However, the upregulation of the short transcript levels observed in Col-0 in response to zinc excess was lost in Sha and NIL-Sha (Fig. 7). In shoots, levels of *AtFRD3*_*tot*_ were strongly increased under Zn deficiency in all genotypes (Supplementary Fig. S8). The *AtFRD3*_*S*_/*AtFRD3*_*L*_ ratio as well as the upregulation of *AtFRD3*_*L*_ transcript levels under zinc deficiency were conserved across all five genotypes (Fig. 7).

## Discussion

Here, we examined the regulation of *FRD3* expression in two closely related Brassicaceae, *A. thaliana* and *A. halleri*, with contrasting metal homeostasis (Verbruggen *et al.*, 2009; Krämer, 2010; Hanikenne and Nouet, 2011).

### *FRD3* is expressed in both roots and shoots

We showed that the spatial pattern of *AtFRD3* promoter activity was conserved in roots and shoots of both *A. thaliana* and *A. halleri*. In roots, the *AtFRD3* promoter was highly active in the pericycle and vascular cylinder in roots (Fig. 2), as described previously (Rogers and Guerinot, 2002; Green and Rogers, 2004; Roschzttardtz *et al.*, 2011). In *A. thaliana* roots, iron chelation by citrate, which depends on FRD3 function, is of critical importance for root-to-shoot mobility of iron in the xylem (Rogers and Guerinot, 2002; Green and Rogers, 2004; Durrett *et al.*, 2007), and FRD3 contributes to zinc tolerance (Pineau *et al.*, 2012).

FRD3-mediated citrate release into the apoplast also contributes to lateral iron transport from xylem to leaf parenchyma and from the phloem to surrounding tissues in old leaves (Roschzttardtz *et al.*, 2011; Schuler *et al.*, 2012). The *AtFRD3* promoter was active in leaf veins and mesophyll cells (Fig. 2). Whereas several studies concluded that *AtFRD3* is not expressed in leaves (Rogers and Guerinot, 2002; Green and Rogers, 2004; Roschzttardtz *et al.*, 2011), others reported *AtFRD3* expression in leaves using either ATH1 microarrays (Supplementary Fig. S9) or quantitative RT-PCR (Talke *et al.*, 2006). We detected *AtFRD3* expression in leaves of both seedlings (Fig. 4A–C) and adult plants (data not shown), but steady-state transcript levels were much lower in leaves than in roots of adult plants. This suggests that *AtFRD3* expression in leaves changes dynamically with developmental stages or environmental conditions, which may account for apparent discrepancies in the literature.

### Two transcript variants and two promoters for *AtFRD3*

The *AtFRD3* gene gives rise to two transcript variants from alternative transcription initiation sites. A full-length *AtFRD3* promoter fragment of approximately 4500bp initiated expression of the two transcripts predominantly in vascular tissues of both *A. thaliana* and *A. halleri* (Fig. 2, Supplementary Fig. S3). By contrast, a truncated *AtFRD3* promoter including the proximal transcription initiation site contained the elements required for cell-type-specific expression only in *A. thaliana* and not in leaves of *A. halleri*. The lack of expression of pAtFRD3_Trunc_ in *A. halleri* leaves could result either from the absence of the required *cis*-elements or from the need for a specific *trans*-factor present only in *A. thaliana* shoots. Moreover, the data suggest that *AtFRD3* transcription is initiated at two core promoters of similar function, one distal and one proximal to the ATG. The presence of TATA boxes ~30bp upstream of both transcription initiation sites supports this hypothesis. Both *AtFRD3* promoter fragments specified constitutive and high expression of the *GUS* gene in both species, at levels similar to *AhFRD3* promoter-driven *GUS* expression (Figs 2 and 3). In *A. thaliana* reporter lines, the *GUS* expression levels generated by the full-length *AtFRD3* promoter were substantially higher than endogenous *FRD3* transcript levels (Fig. 3B), suggesting that this promoter fragment does not contain all *cis*-elements required to control *AtFRD3* expression. In *A. thaliana*, *AtFRD3* transcription may be downregulated by an as-yet-unidentified repressive *cis*-regulatory element that was not included in the constructs tested in our study. Further work will be required to identify this putative repressor element. The full-length *AtFRD3* promoter fragment (4506bp) included the entire intergenic region between *AtFRD3* and the 5′ upstream gene (At3g08490), indicating that the repressor element is located either at a greater distance upstream from *AtFRD3*, within transcribed regions of *AtFRD3* (coding sequence, introns, 3′UTR), or in 3′ sequences further downstream from the gene (Ito *et al.*, 2003; Baek *et al.*, 2011; Cao *et al.*, 2014). It is possible that different *cis* regions of the *AtFRD3* gene interact in regulating steady-state *AtFRD3* transcript levels and that absence of these interactions in our promoter–reporter constructs contribute to higher-than-expected *GUS* transcript levels in *A. thaliana*.

### Hidden regulation of *AtFRD3* by zinc

Whereas the total transcript levels of *FRD3* in *A. thaliana* roots (Fig. 4A) appeared unresponsive to zinc, we showed here that the steady-state levels of the two transcript variants of *AtFRD3* responded differentially to zinc supply (Fig. 4B, C). In control conditions, *AtFRD3*_*L*_ was the predominant form, whereas *AtFRD3*_*S*_ levels were upregulated upon zinc excess in roots (Fig. 4B–D). In shoots, total *AtFRD3* transcript levels increased under zinc deficiency (2.5-fold), which was attributable to *AtFRD3*_*L*_ (Fig. 4A, B). In addition, the regulation of the *AtFRD3* transcript variants differed between zinc-tolerant and zinc-sensitive *A. thaliana* accessions, with specific responsiveness to zinc excess (*FRD3*_*S*_) and a conserved response to zinc deficiency (*FRD3*_*L*_) (Fig. 7, Supplementary Fig. S8). Thus, in both accessions the responses to zinc excess and zinc deficiency were distinct, suggesting distinct regulatory mechanisms for each variant transcript.

As exposure to excess zinc can result in secondary iron deficiency (Mendoza-Cozatl *et al.*, 2008; Fukao *et al.*, 2011; Shanmugam et al., 2011, 2013; Pineau *et al.*, 2012), it may be postulated that the zinc-dependent regulation of *FRD3* reported here was an iron-deficiency response. However, the zinc concentration used was relatively low compared with previous studies (Shanmugam *et al.*, 2011; Pineau *et al.*, 2012), avoiding major toxicity effects on seedlings. This treatment did not cause major alterations in iron concentrations in plants (Supplementary Fig. S5A), suggesting a possible direct regulation of *AtFRD3* in response to zinc. Similarly, the zinc-deficiency treatment did not alter iron concentrations in shoot tissues (Supplementary Fig. S5A). The specific regulation of *AtFRD3*_*L*_ observed upon zinc deficiency in shoots probably represents a direct response to this condition. The functional significance of the upregulation of *AtFRD3*_*L*_ by zinc deficiency in shoots remains unclear.

### Alternative transcription initiation sites, transcript stability, and impact on translation

The *AtFRD3* gene displays complex regulation: two short-lived transcripts, which differ in their 5′UTR only, are initiated from alternative transcription initiation sites and have different translation efficiencies. Large-scale studies in *A. thaliana* have revealed that, across all transcripts encoded in the genome, mRNA stability varies enormously, with half-life values ranging from 12min to 24h (with a mean of 6h and a median of 2h) (Gutiérrez *et al.*, 2002; Narsai *et al.*, 2007). The *AtFRD3* transcript variants group among the transcripts of low stability. In *A. thaliana*, this would allow rapid shifts from one variant to the other in response to changes in zinc status. A low stability of endogenous *AtFRD3* transcripts may partly contribute to the observed differences between *GUS* transcript levels (~30min half-life; data not shown) and endogenous *FRD3* transcript levels in *A. thaliana* reporter lines (Fig. 3B).

The production of alternative transcripts from a single gene is a widespread phenomenon in eukaryotes and is responsible for proteome diversity. Alternative transcripts can be generated through two types of event: alternative splicing and alternative transcription initiation sites. Both types of event are highly frequent in mammals (Davuluri *et al.*, 2008; Pan *et al.*, 2008; Ma *et al.*, 2009; Shabalina *et al.*, 2010) and plants (Tanaka *et al.*, 2009; Filichkin *et al.*, 2010).

We could not directly evaluate protein accumulation *in planta* in response to metal treatments as in our hands it was neither possible to raise antibodies against FRD3 nor to detect expression of FRD3 proteins tagged with GFP or a FLAG tag in tissues of either transiently or stably transformed plants (data not shown). Instead, we assessed the functional impact of alternative 5′UTRs on transcript levels, transcript stability, and translation using GFP reporter constructs (Dvir *et al.*, 2013; Kim *et al.*, 2014; Remy *et al.*, 2014). The 5′UTR of *AtFRD3*_*S*_ transcripts gave rise to ~2.5-fold higher protein levels inferred from relative GFP fluorescence compared with *AtFRD3*_*L*_ suggesting a more efficient translation (Fig. 6). Differential translation of the two transcript variants probably amplified the effect of the differential transcriptional changes incurred by the two variants upon zinc treatment (Fig. 4). This complex regulation affords increased protein accumulation, and probably increased citrate efflux into the apoplast, upon zinc excess, even when total *AtFRD3* transcript levels remain unchanged. Increased expression of *AtFRD3*, as observed in a *nicotianamine synthase* quadruple (*nas4x-2*) mutant, indeed resulted in increased citrate levels in the xylem sap (Schuler *et al.*, 2012). Similarly, 5′UTR variants of *AtZIF2* resulting from alternative splicing promote distinct translation efficiencies and contribute differentially to zinc tolerance (Remy *et al.*, 2014).

There are several mechanisms by which the 5′UTR can influence translation, via the presence of sequence elements controlling ribosome binding and translation initiation, such as the presence of micro-open reading frames (micro-ORFs) (Alatorre-Cobos *et al.*, 2012) or secondary structures (Bugaut and Balasubramanian, 2012; Pichon *et al.*, 2012). In *A. thaliana*, efficient translation initiation requires a short 5′UTR (between 40 and 175 nt), the absence of micro-ORFs, a specific sequence context around the AUG, and a high A content (Kawaguchi and Bailey-Serres, 2005). We detected no micro-ORF in *AtFRD3* transcript variants, and both share a similarly low G+C content with 37.5% for *AtFRD3*_*L*_ and 34.2% for *AtFRD3*_*S*_. We did not detect the presence of a TAGGGTTT *cis* element, which confers higher translatability to mRNAs in *A. thaliana* (Liu *et al.*, 2012). After splicing, both *AtFRD3*_*L*_ and *AtFRD3*_*S*_ 5′UTRs share a similar size. Prediction of the secondary structure of the two transcript variants using Centroidfold (Sato *et al.*, 2009) suggested the presence of a disorganized loop structure in the 5′UTR of *AtFRD3*_*L*_, which is absent in *AtFRD3*_*S*_ (Supplementary Fig. S7). This difference in secondary structure may account for the distinct translation determined by the two variant 5′UTRs.

### *A. halleri* maintains an ancestral highly expressed and translation-efficient *FRD3* transcript

In contrast to *A. thaliana*, the expression of *A. halleri FRD3* was not regulated in response to alterations in zinc supply (Fig. 4E), as shown previously (Talke *et al.*, 2006). The spatial pattern of expression of *AhFRD3* was conserved compared with *AtFRD3* in roots and shoots of *A. halleri* and *A. thaliana* (Fig. 2). Expression of *AhFRD3* in shoots corresponded to expectations based on transcript profiling in *A. halleri* (Talke *et al.*, 2006). *AhFRD3* was also expressed in anthers and in the embryo of *A. thaliana* (data not shown), as reported recently for *AtFRD3* (Roschzttardtz *et al.*, 2011).

Whereas the transcription of two *FRD3* transcript variants appears to be a shared feature in Brassicaceae, *A. halleri* constituted an exception with a single short *FRD3* transcript (Fig. 1, Supplementary Fig. S1). The transcription initiation sites of *AhFRD3* and *AtFRD3*_*S*_ were evolutionarily conserved (Fig. 1), with both transcripts displaying highly similar 5′UTRs (94.2% identity and a low G+C content of ~34%) that determined equally efficient translation (Fig. 6, Supplementary Fig. S6). *AhFRD3* transcripts, however, displayed slightly higher transcript stabilities (~1.6-fold) in comparison with *AtFRD3*_*S*_ transcripts (Fig. 5). In addition, as suggested above based on our finding that the *AtFRD3* promoter fragment examined in this study directs high constitutive expression, *AtFRD3* transcription may be subject to repression through an as-yet-unidentified *cis*-regulatory element in *A. thaliana*. Parsimoniously, we suggest that any repressor element present at the *AtFRD3* locus has been lost at the *AhFRD3* locus, resulting in constitutively high expression of *AhFRD3* in *A. halleri*.

Based on our observations and the considerations detailed above, we propose the following hypothetic scenario, with two loss-of-function events, for the evolution of high expression of *FRD3* in *A. halleri*. The ancestral short transcript variant (*FRD3*_*S*_), with a short intronless, translation-efficient 5′UTR and pre-existing regulatory responsiveness to zinc excess, was favoured and retained during the evolutionary history of *A. halleri*. Together with a derepression through mutations in *cis* and enhanced transcript stability, this allowed constitutively high expression of *FRD3* in *A. halleri* (Talke *et al.*, 2006). Further work will be required to identify the *cis*-regulatory elements responsible for *AhFRD3* high promoter activity.

In *A. halleri* and in zinc-tolerant *A. thaliana* genotypes, constitutively higher expression of the *FRD3*_*S*_ transcripts appeared to be associated with increased zinc tolerance (Figs 4E and 7) and possibly with increased metal accumulation. Indeed, constitutive expression of *FRD3*_*S*_ may be responsible for the higher accumulation of zinc observed in shoots of Bay-0 (Pineau *et al.*, 2012). In *A. halleri*, increased FRD3-mediated release of citrate into the xylem might also contribute to zinc mobility towards the shoot and participate in hyperaccumulation. Indeed, a recent study showed that zinc is predominantly bound to organic acids, including citrate, in the xylem sap of *A. halleri* (Cornu *et al.*, 2015). Further work, including targeted silencing of *FRD3* in *A. halleri*, will be required to test the function of *FRD3* in zinc tolerance.

So far, higher expression levels of metal homeostasis genes have been linked to gene copy number expansion and/or (*cis*-)regulatory changes in *A. halleri* compared with *A. thaliana* (Talke *et al.*, 2006; Hanikenne *et al.*, 2008; Shahzad *et al.*, 2010). In contrast, *FRD3* is a single-copy gene, and our data suggest an original path for the evolution of high expression of *FRD3* in *A. halleri* building on complex regulation mechanisms already present in the non-hyperaccumulating ancestor of *A. halleri*. This suggests that a variety of types of molecular alterations underlie this extreme naturally selected trait.

## Supplementary data

Supplementary data are available at *JXB* online.

Supplementary Fig. S1. Distribution of the *FRD3*_*L*_ and *FRD3*_*S*_ transcript variants in *Arabidopsis* relatives.

Supplementary Fig. S2. *FRD3* promoter *GUS* reporter constructs.

Supplementary Fig. S3. Detection of *GUS* transcripts in reporter lines.

Supplementary Fig. S4. Metal ion concentrations in *A. thaliana* and *A. halleri* plant tissues.

Supplementary Fig. S5. Transcript levels of *ZIP4* in response to zinc deficiency and excess in *A. thaliana* and *A. halleri*.

Supplementary Fig. S6. Effect of *FRD3* variant 5′UTRs onto the translation of a downstream fused GFP-encoding transcript.

Supplementary Fig. S7. Secondary structure prediction of the *AtFRD3* transcript variants.

Supplementary Fig. S8. Differential transcriptional regulation of *AtFRD3* in zinc-tolerant and zinc-sensitive *A. thaliana* genotypes.

Supplementary Fig. S9. *FRD3* expression profile in *A. thaliana*.

Supplementary Table S1. List of primers used to generate genetic constructs.

Supplementary Table S2. List of RT-PCR primers.

Supplementary Table S3. List of real-time RT-PCR primers.

Supplementary Data

## Abbreviations:

Col-0: Columbia-0
GFP: green fluorescent protein
GUS: β-glucuronidase
LUC: luciferase
micro-ORF: micro-open reading frame
RACE: rapid amplification of cDNA ends
RT-PCR: reverse transcription PCR
UTR: untranslated region.
