# Supplementary material for: Zinc triggers a complex transcriptional and post-transcriptional regulation of the metal homeostasis gene FRD3 in Arabidopsis relatives
Source: J Exp Bot. 2015 Apr 21;66(13):3865–78. doi: 10.1093/jxb/erv188 (PMC4473987; doi:10.1093/jxb/erv188)
Supplement: Supplementary Data [file supp_66_13_3865__index.html]

Zinc triggers a complex transcriptional and post-transcriptional regulation of the metal homeostasis gene FRD3 in Arabidopsis relatives — Supplementary Data 

# Zinc triggers a complex transcriptional and post-transcriptional regulation of the metal homeostasis gene *FRD3* in *Arabidopsis* relatives

## Supplementary Data

Data files

**Files in this Data Supplement:**

- Supplementary Data - Supplementary Data
