# Supplementary material for: Zinc triggers a complex transcriptional and post-transcriptional regulation of the metal homeostasis gene FRD3 in Arabidopsis relatives
Source: J Exp Bot. 2015 Apr 21;66(13):3865–78. doi: 10.1093/jxb/erv188 (PMC4473987; doi:10.1093/jxb/erv188)
Supplement: Supplementary Data [file supp_erv188_erv188_Charlier_etal_supplementary_data.pdf]

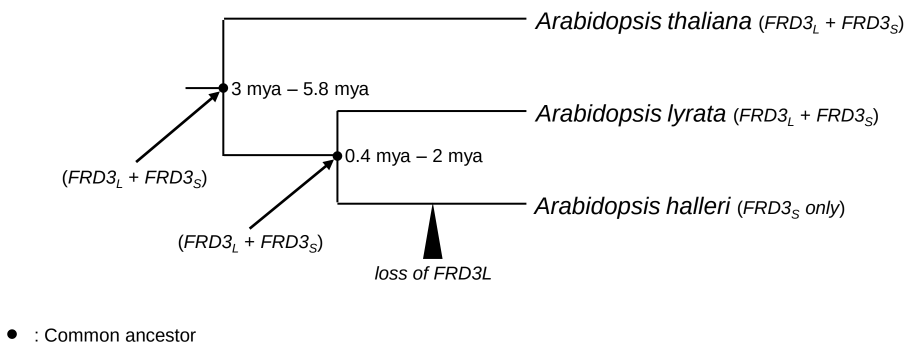

Fig. S1. Distribution of the *FRD3*$_L$ and *FRD3*$_S$ transcript variants in Arabidopsis relatives.

In this schematic representation of the phylogenetic relationship between *A. thaliana*, *A. lyrata* and *A. halleri*, the estimated times of divergence of the species are indicated in million years ago (mya) according to Yogeeswaran et al. (2005), Clauss and Koch (2006) and Roux et al. (2011).

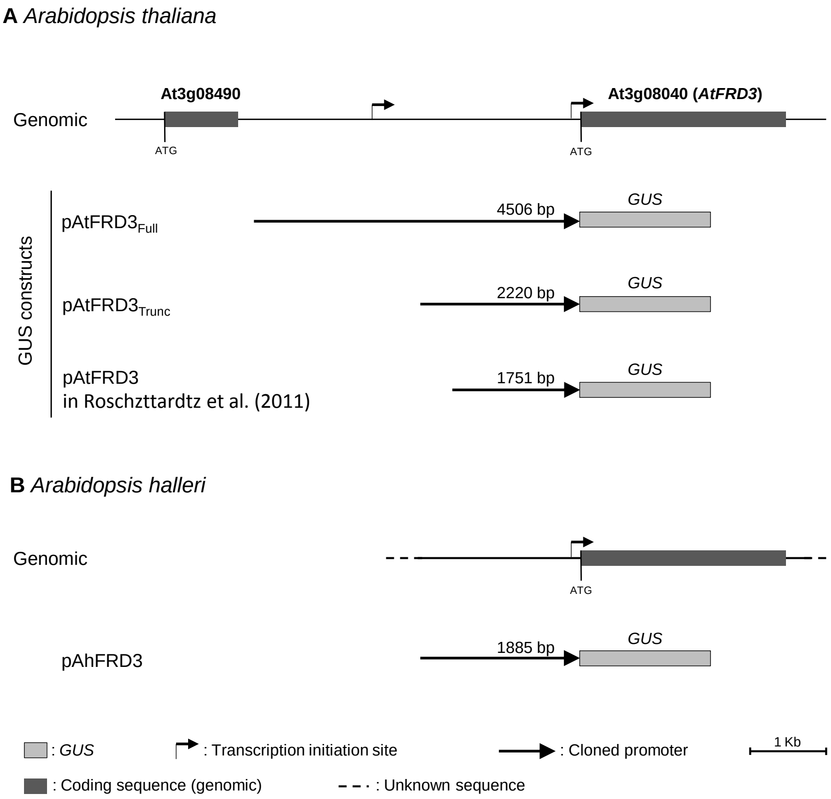

Fig. S2. *FRD3* promoter GUS reporter constructs.

Organization of the *FRD3* genomic loci and cloned promoter fragments of **(A)** *A. thaliana* and **(B)** *A. halleri*. The full (pAtFRD3$_{\text{Full}}$) and truncated (pAtFRD3$_{\text{Trunc}}$) used in this study are compared to the construct used in a previous study (Roschzttardtz et al., 2011), and to the *A. halleri FRD3* (pAhFRD3) promoter.

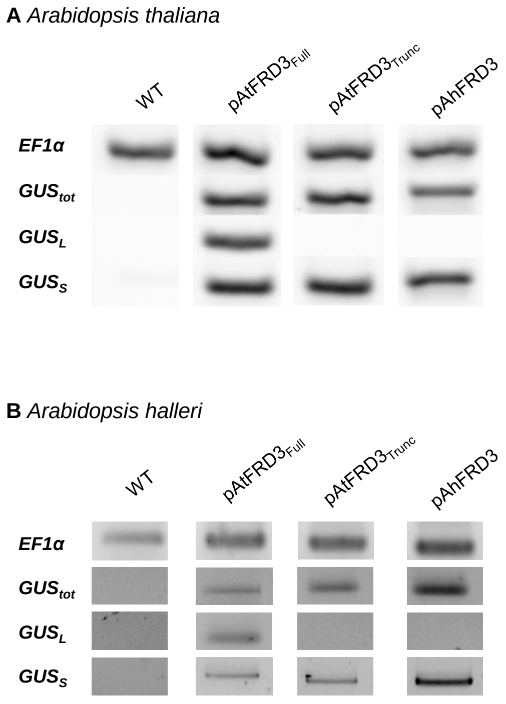

Fig. S3. Detection of *GUS* transcripts in reporter lines.

Total *GUS* transcripts (*GUS*$_{tot}$) and transcripts including the long (*GUS*$_{L}$) or the short (*GUS*$_{S}$) *FRD3* 5'UTR, respectively, were detected by qualitative RTPCR in **(A)** 12-day-old *A. thaliana* seedlings and **(B)** roots of 4.5 week-old *A. halleri* plants grown hydroponically expressing the *GUS* reporter gene under the control of a full (pAtFRD3$_{\mathrm{Full}}$) and a truncated (pAtFRD3$_{\mathrm{Trunc}}$) *A. thaliana FRD3* promoter, or the *A. halleri FRD3* (pAhFRD3) promoter. Wild-type (WT) plants and *EF1α* were used as control genotype and gene, respectively.

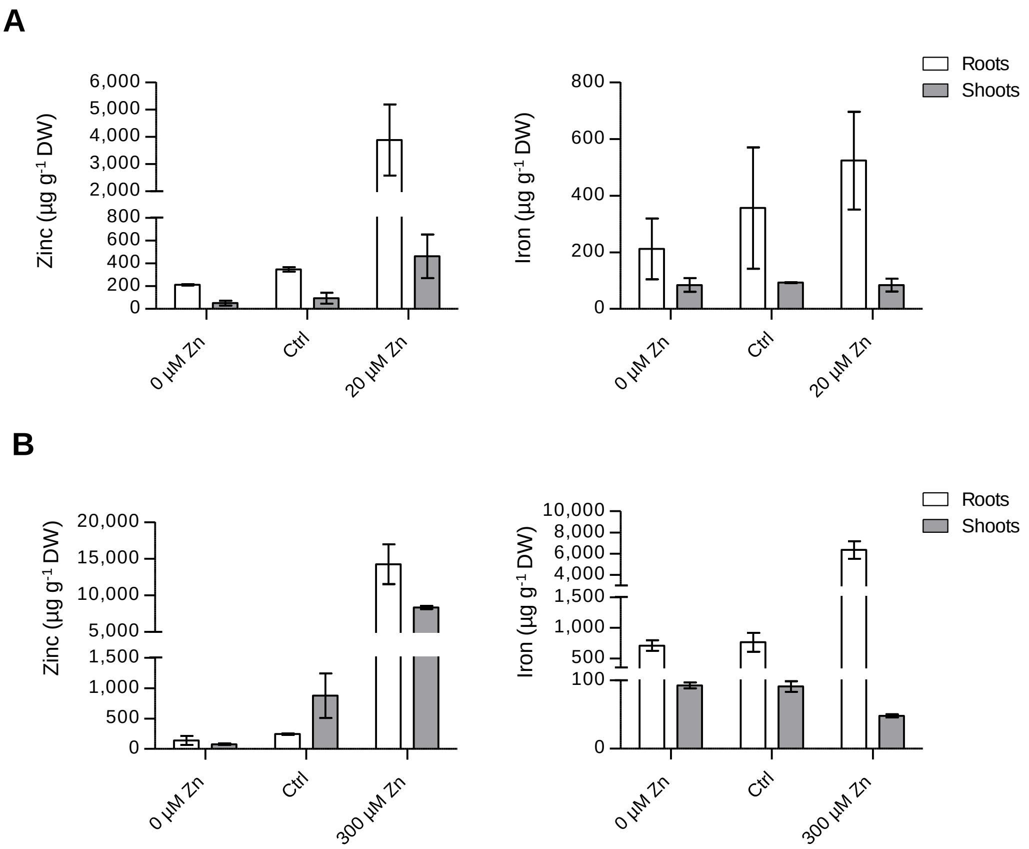

Fig. S4. Metal ion concentrations in *A. thaliana* and *A. halleri* plant tissues.

(A) Zinc and iron concentrations in roots and shoots of *A. thaliana* upon zinc deficiency (0 µM Zn), control conditions (Ctrl, 1 µM Zn) and zinc excess (20 µM Zn). Values are mean ± SEM of 2 independent experiments. Independent experiments included pool of at least 25 *A. thaliana* seedlings grown on Hoagland agar plates for each condition.
(B) Zinc and iron concentrations in roots and shoots of *A. halleri* upon zinc deficiency (0 µM Zn), control conditions (Ctrl, 5 µM Zn) and zinc excess (300 µM Zn). Values are mean ± SEM of 2 independent experiments. Independent experiments included pool of at least 6 *A. halleri* plants grown hydroponically in Hoagland medium for each condition.
Metal ion concentrations were determined by ICP-AES. DW: Dry Weight.

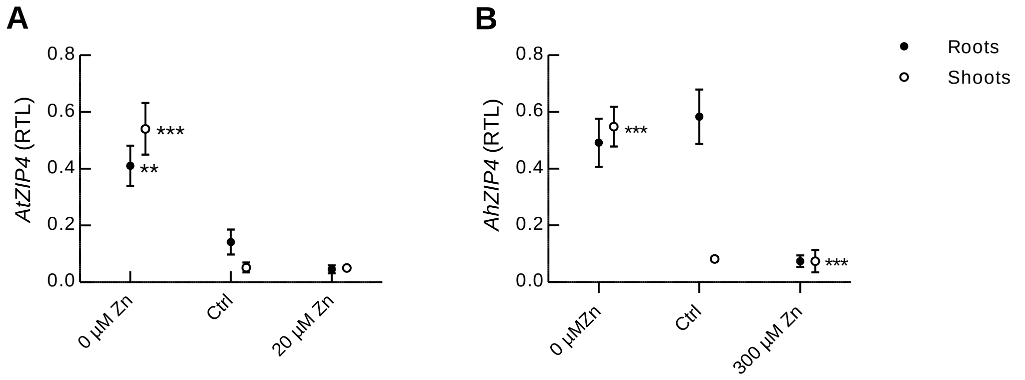

Fig. S5. Transcript levels of *ZIP4* in response to zinc deficiency and excess in *A. thaliana* and *A. halleri*.

(A) *AtZIP4* transcript levels in roots and shoots of *A. thaliana* upon zinc deficiency (0 µM Zn), control conditions (Ctrl, 1 µM Zn) and zinc excess (20 µM Zn). Values are mean ± SEM of 3 independent experiments. Independent experiments included pools of at least 25 *A. thaliana* seedlings grown on solidified Hoagland medium for each condition.
(B) *AhZIP4* transcript levels in roots and shoots of *A. halleri* upon zinc deficiency (0 µM Zn), control conditions (Ctrl, 5 µM Zn) and zinc excess (300 µM Zn). Values were normalized to *EF1α* and an inter-run calibrator. Values are mean ± SEM of 2 independent experiments. Independent experiments included pools of at least 6 *A. halleri* plants grown hydroponically in Hoagland medium for each condition.
Values were normalized to *EF1α* and an inter-run calibrator. The inter-run calibrator differed for each species, and thus transcript levels can only be compared within species. ** $p < 0.01$, *** $p < 0.001$ according a one-way ANOVA followed by Dunnett's test for multiple comparison of means. RTL: Relative Transcript Level.

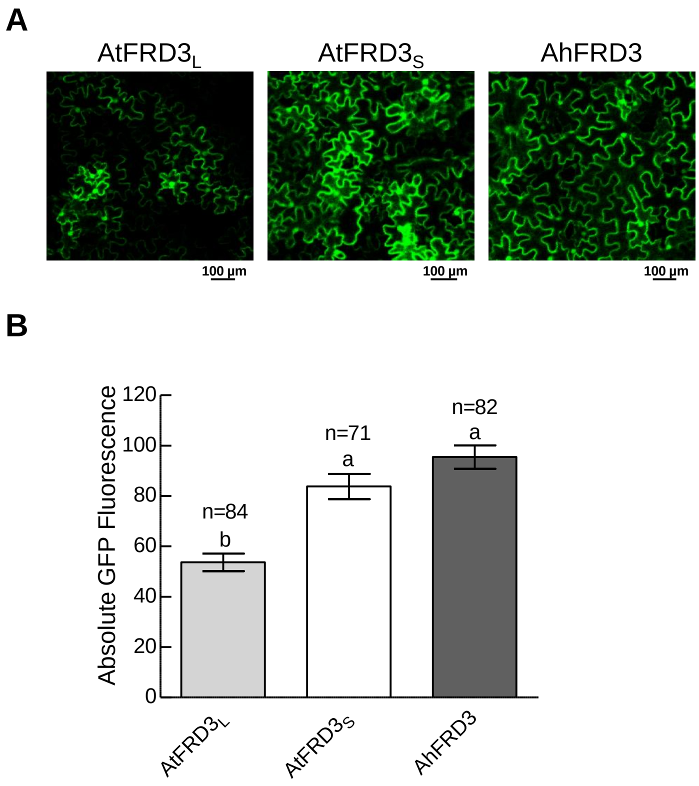

Fig. S6. Effect of *FRD3* variant 5'UTRs onto the translation of a downstream fused GFP-encoding transcript.

AtFRD3$_L$, *AtFRD3*$_S$ and *AhFRD3* 5'UTR fused to the GFP coding sequence were transiently expressed in tobacco leaves under the control of a 35S promoter.
(A) Confocal imaging of GFP fluorescence in representative leaf fragments. Images were recorded with identical settings, disallowing saturation of the GFP signal in any of the samples.
(B) GFP fluorescence was quantified by confocal imaging in the nucleus of leaf epidermal cells. Values are mean ± SEM (n = 71 to 84 nuclei from at least 3 independent transient transformations). Letters above histogram bars indicate significantly different values ($p < 0.05$), according to a Kolmogorov-Smirnov (with Dallal-Wilkinson-Lilliefor p value) normality test, followed by a Mann-Whitney test for significance.

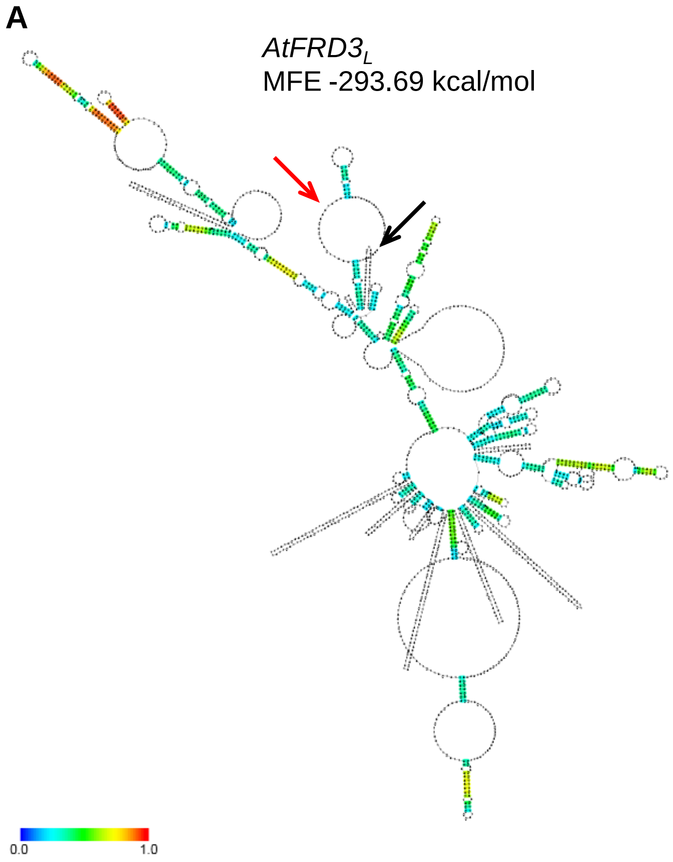

Fig. S7. Secondary structure prediction of the *AtFRD3* transcript variants.

Centroifold was used to predict the secondary structure of the full-length mature *AtFRD3*$_L$ **(A)** and *AtFRD3*$_S$ **(B)** transcripts. A putative secondary structure element found only in the 5' extremity of *AtFRD3*$_L$ is indicated by a red arrow. The black arrows indicate the 5' extremity of the transcripts. The color code indicates base pairing probabilities (red – high, blue – low) and the minimum free energy (MFE) of the entire structures is indicated.

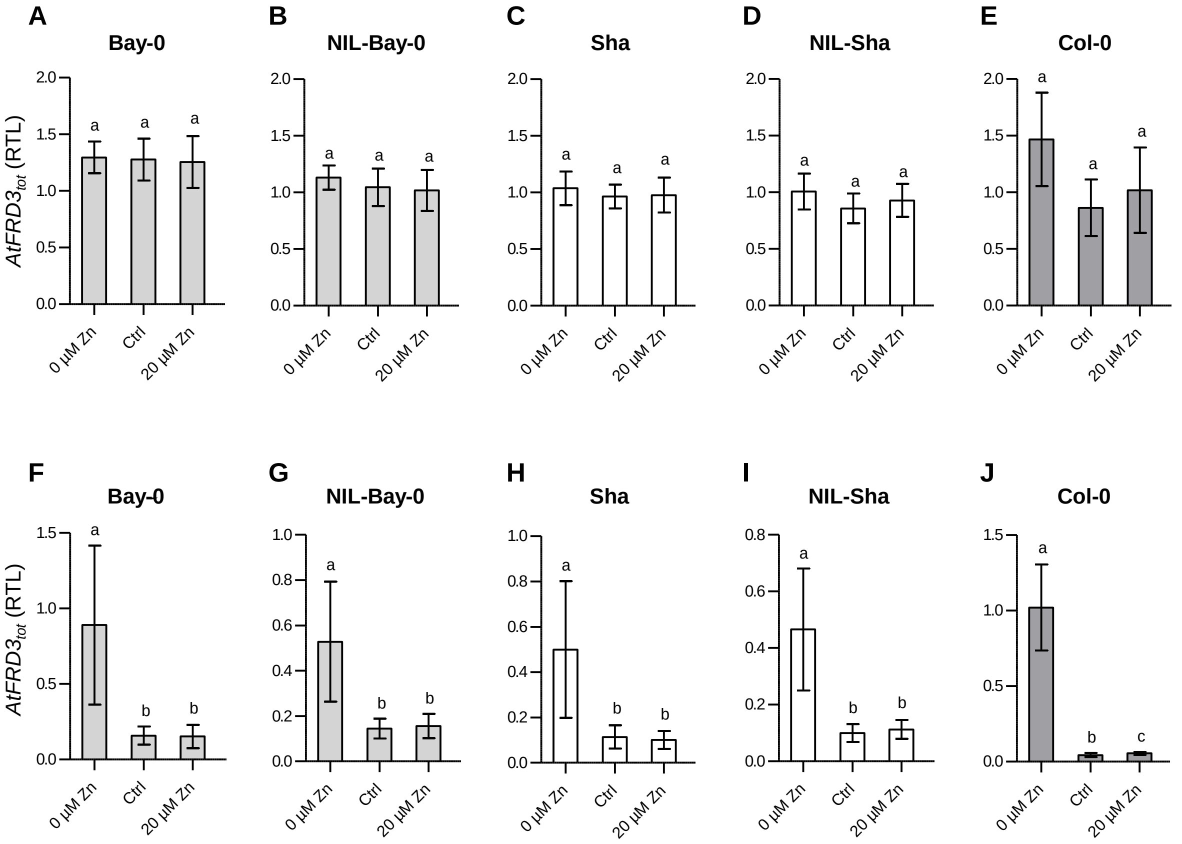

Fig. S8. Differential transcriptional regulation of *AtFRD3* in zinc-tolerant and zinc-sensitive *A. thaliana* genotypes.

Steady-state levels were determined for total *AtFRD3* (*AtFRD3tot*) transcript in roots **(A-E)** and shoots **(F-J)** of 21-day-old *A. thaliana* seedlings cultivated under control conditions (Ctrl, 1 µM Zn) and upon zinc deficiency (0 µM Zn) or zinc excess (20 µM Zn) for 17 days. Bay-0 and NIL-Bay are zinc-tolerant genotypes whereas Sha and NIL-Sha are zinc-sensitive genotypes (Pineau *et al.*, 2012). Col-0 was included as a reference genotype. Values were normalized to *EF1α* and an inter-run calibrator. Values are mean ± SEM of 2 independent experiments. Independent experiments included pools of at least 25 seedlings per genotype and treatment. Letters above histogram bars indicate significantly different values ($p < 0.05$) within treatments according to a one-way ANOVA and Dunnett's test for multiple comparison of means. RTL: Relative Transcript Level.

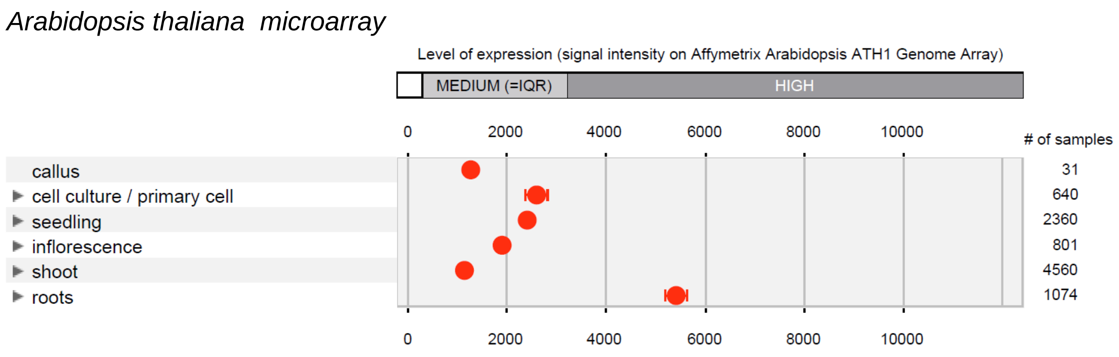

Fig. S9. *FRD3* expression profile in *A. thaliana*.

FRD3 expression profile in *A. thaliana* as described in Genevestigator, based on 9466 sample micro-arrays for 127 anatomical parts.
Data retrieved the 07/01/2015 on https://www.genevestigator.com/gv/plant.jsp.

Table S1. List of primers used to generate genetic constructs.

<table>
<tr><th>Target</th><th>Forward (3’-5’)</th><th>Reverse (5’-3’)</th><th></th></tr>
<tr><td>pAtFRD3$_{Full}$</td><td>CACCAACAATATAGACCCACTTGCGGC</td><td rowspan="3">AGCAAGATCATCACCAGTTTCCG</td><td>Cloning of pAtFRD3$_{Full}$</td></tr>
<tr><td>pAtFRD3$_{Trunc}$</td><td>CACCCAACATAGCAAAAGATGATGTACACA</td><td>Cloning of pAtFRD3$_{Trunc}$</td></tr>
<tr><td>pAhFRD3</td><td>CACCGAGTATTATCTTGTCACCATGTTTTATTCG</td><td>Cloning of pAhFRD3</td></tr>
<tr><td>GFP</td><td>GTGGTACCATGGTGAGCAAGGGCGAGGAG</td><td>ACACTTAATTAATTACTTGTACAGCTCGTCCATGC</td><td>Fusion of GFP with 5’UTR</td></tr>
<tr><td>AtFRD3$_L$ 5’UTR</td><td>ACACGGCGCGCCACTCACTCATCACTACTCATCTC</td><td rowspan="2">ACACGGTACCAGATCTTTTTCCTCTGTAACTGTCTC</td><td>Fusion of AtFRD3$_L$ 5’UTR with GFP for 35S:AtFRD3L 5’UTR-GFP:tNOS construction</td></tr>
<tr><td>AtFRD3$_S$ 5’UTR</td><td rowspan="2">ACACGGCGCGCCACTATAAACGTTCCTTTTGCTTCC</td><td>Fusion of AtFRD3$_S$ 5’UTR with GFP for 35S:AtFRD3S 5’UTR-GFP:tNOS construction</td></tr>
<tr><td>AhFRD3 5’UTR</td><td>ACACGGTACCAAATCTTTTCCTTCGTAACTGTCTC</td><td>Fusion of AhFRD3 5’UTR with GFP for 35S:AhFRD3 5’UTR-GFP:tNOS construction</td></tr>
<tr><td>LUC</td><td>ACACGGCGCGCCATGGAAGACGCCAAAAACATAAAAG</td><td>ACACGAGCTCTTACAATTTGGACTTTCCGCCC</td><td>Construction of plasmid 35S:LUC:tNOS</td></tr>
<tr><td>GFP</td><td>CGGGCGCGCCATGGTGAGCAAGGGCGAGGAG</td><td>ACACTTAATTAATTACTTGTACAGCTCGTCCATGC</td><td>Construction of plasmid 35S:LUC:tNOS</td></tr>
</table>

Specific sequence features are highlighted in primer sequences: *Kpn*I restriction site (GGTACC); *Asc*I restriction site (GGCGCGCC); *Pac*I restriction site (TTAATTAA); CACC sequence allowing directional cloning in the GATEWAY entry vector pENTRD.

Table S2. List of RT-PCR primers.

**Target**	**Forward** (3'-5')	**Reverse** (5'-3')	**Nb of cycles**
AtFRD3tot	GTTTATAGCAGCCACGCAGCC	CTAGGAAGATGAAGAGGATGATCGTC	32
AtFRD3L	CACTACTCATCTCAAGTTCACGTGAC	GCTGAACCGCTCCTAAACGC	32
AtFRD3S	CCGATTCTTCGAAACACTTATTGAT	GCTGAACCGCTCCTAAACGC	32
At/AhEF1α	TAAGGATGGTCAGACCCGTGA	GAGACTCGTGGTGCATCTCAAC	28
AtZIP4	GAACCGCCGGTTTCTTCAA	ATCGGTCACTCTTTCCCCAAC	32
AhFRD3	CCGATTCTTCGAAACACTTATTGAT	GCTGAGCCGCTCCTAAACGC	31
AhZIP4	ACCGTCGGTTCCTTCAAACC	CGTTGACACTTTCCCCAACCAC	32
GFP	TGAAGTTCATCTGCACCACCG	CCTTGATGCCGTTCTTCTGCT	25
HygB	ATTGGGGAGTTTAGCGAGAGC	CTCCAGTCAATGACCGCTGTT	25
GUS	ACAAAAACCACCCAAGCGTG	CATATCCAGCCATGCACACTG	32
GUS_L	CACTACTCATCTCAAGTTCACGTGAC	CGTTAAAACTGCCTGGCACAG	32
GUS_S	CCGATTCTTCGAAACACTTATTGAT	CGTTAAAACTGCCTGGCACAG	32

Table S3. List of real-time PCR primers.

**Target**	**Forward** (3'-5')	**Reverse** (5'-3')
AtEF1a	TGAGCACGCTCTTCTTGCTTTCA	GGTGGTGGCATCCATCTTGTTACA
AtFRD3$_{tot}$	CGATATTCCCACTTGTGAGCC	TTCTCCATCGTGTCTTCCTCTG
AtFRD3$_{L}$	CACTACTCATCTCAAGTTCACGTGAC	GGGATTGGCTTCTTCACCG
AtFRD3$_{S}$	CCGATTCTTCGAAACACTTATTGAT	GGGATTGGCTTCTTCACCG
AtFER1	TTCCAACGATGGCCTCAAAC	ACTTTCCTGGAGAAGCCGAGA
AtSAND	GTTTGCGCGTCTGGTGTCTTA	GGATGGAGAGACGCTTTCTGTG
AhFRD3	TGTGGCAGAGGAAGACACGAT	TCTGCATGAACAAGACTGGCTT
GFP	GAGCAAAGACCCCAACGAGAA	ACTTGTACAGCTCGTCCATGCC
AtFRD3L 5'UTR:GFP	CACTACTCATCTCAAGTTCACGTGAC	ACTTGTACAGCTCGTCCATGCC
AtFRD3S 5'UTR:GFP	CCGATTCTTCGAAACACTTATTGAT	ACTTGTACAGCTCGTCCATGCC
AhFRD3 5'UTR:GFP	TGTGGCAGAGGAAGACACGAT	ACTTGTACAGCTCGTCCATGCC
